# Exosomes in the Regulation of Vascular Endothelial Cell Regeneration

**DOI:** 10.3389/fcell.2019.00353

**Published:** 2020-01-09

**Authors:** Jugajyoti Baruah, Kishore K. Wary

**Affiliations:** ^1^Department of Psychiatry, Harvard Medical School, Boston, MA, United States; ^2^Angiogenesis and Brain Development Laboratory, Division of Basic Neuroscience, McLean Hospital, Belmont, MA, United States; ^3^Department of Pharmacology, The University of Illinois at Chicago, Chicago, IL, United States

**Keywords:** angiogenesis, exosomes, endothelial cells, regeneration, rejuvenation

## Abstract

Exosomes have been described as nanoscale membranous extracellular vesicles that emerge from a variety of cells and tissues and are enriched with biologically active genomic and non-genomic biomolecules capable of transducing cell to cell communication. Exosome release, and exosome mediated signaling and cross-talks have been reported in several pathophysiological states. Therefore, exosomes have the potential to become suitable for the diagnosis, prognosis and treatment of specific diseases, including endothelial cell (EC) dysfunction and regeneration. The role of EC-derived exosomes in the mechanisms of cardiovascular tissue regenerative processes represents currently an area of intense research activity. Recent studies have described the potential of exosomes to influence the pathophysiology of immune signaling, tumor metastasis, and angiogenesis. In this review, we briefly discuss progress made in our understanding of the composition and the roles of exosomes in relation to EC regeneration as well as revascularization of ischemic tissues.

## Introduction

Exosomes are nanometer (30–100 nm) sized membranous vesicles originating during the formation of multivesicular bodies (MVBs) (Dignat-George and Boulanger, 2011; Ribeiro et al., 2013; Boulanger et al., 2017; Théry et al., 2018). Biochemically, exosomes are characterized by the presence of CD63, CD14, TSG101, heat shock protein and flotillin; these exosomes can be sedimented at 120,000 × g (Théry et al., 2006; Vlassov et al., 2012; Kowal et al., 2016). Exosomes and apoptotic bodies are broadly classified as extracellular vesicles; in contrast to exosomes, apoptotic bodies are larger (500–5000 nm) (Caruso and Poon, 2018), and they sediment at 1,200–10,000 × g (Théry et al., 2006). Exosomal biogenesis occurs in the endosomal pathway and is characterized by biochemical properties (Table 1), whereas apoptotic bodies are characterized by membrane blebbing and their unique cell surface markers, e.g., phosphatidylserine and Annexin-V (Henson et al., 2001). Exosomes are usually released into the extracellular space when MVBs fuse with the plasma membrane, and exosomes can transport lipids, mRNAs and proteins that can alter cellular behavior in a paracrine or autocrine manner (Sahoo and Losordo, 2014; Kishore and Khan, 2016). Depending on the tissue microenvironment, and the exosome content, these vesicles mediate an array of cellular functions (Ribeiro et al., 2013). A classic example of an altered tissue microenvironment is the maintenance and repair of tissues in response to injury. Studies are beginning to document cell-cell signaling events that mediate restorative functions in the tissue microenvironment. In this context, the mechanisms of exosome uptake by target cells might be mediated by more than one mechanism. In cultured cells, exosome uptake can occur through: (a) clathrin-dependent endocytosis, (b) caveolae-dependent endocytosis, (c) phagocytosis, and (d) micropinocytosis (McKelvey et al., 2015). Whether exosome uptake by target cells is a physiologically regulated process remains incompletely understood.

**TABLE 1:** Biochemical properties of apoptosis, exosomes and MVBs.

	**Size (nm)**	**Morphology**	**Sedimentation speed**	**Origin**	**Mechanism of formation**	**Known pathways**
Apoptotic bodies	30–100	Heterogeneous	1,200–10,000 × g	Plasma membrane	Budding from the plasma membrane	Apoptotic pathway
Exosomes	30–100	Cup-shaped	100,000–120,000 × g	Multivesicular body (MVB)	Exocytosis of MVB	ESCRT-dependent, Tetraspanin, ceramide
Microvesicles	100–1000	Heterogeneous	100,000–200,000 × g	Plasma membrane	Budding from the plasma membrane	Ca^++^-dependent, stimuli- and cell-dependent

Tissue repair mechanisms entail effective endothelial cell (EC) regeneration and reestablishment of blood flow in damaged and ischemic tissues. To accomplish this repair process, ECs that form the innermost linings of the blood vessels undergo regeneration and angiogenesis to support the restoration of tissue homeostasis (Carmeliet, 2005; Liu et al., 2019; Miyagawa et al., 2019; Williams and Wu, 2019). EC regeneration is a complex biological process that include EC migration, EC survival, rapid proliferation, tube formation, and ultimately reperfusion of injured tissues to restore homeostasis of the tissue microenvironment (Carmeliet, 2005; Bentley and Chakravartula, 2017; McDonald et al., 2018; Liu et al., 2019; Williams and Wu, 2019). Although several studies have attempted to understand the process of EC or vascular regeneration, the molecular mechanism that drives this process remains incompletely understood. Given the biological properties of exosomes and the events that they can regulate, the idea that exosomes derived from various cell types, including ECs themselves in the damaged tissue niche, can modulate EC regeneration remains an active area of research (Ibrahim et al., 2014; Li et al., 2016; Abid Hussein et al., 2017; Balbi et al., 2017; Adamiak and Sahoo, 2018; Dougherty et al., 2018; Ju et al., 2018; Bian et al., 2019; Cheng et al., 2019). In support of this notion, we describe a compendium of studies conducted over the past decade that highlight both EC- and non-EC derived exosomal molecular cargoes which drive this regenerative process. The idea and the discussions that exosomes might provide therapeutic benefit in the settings of ischemic cardiovascular diseases involving physiological injuries that might otherwise transition to disease states, should be rewarding efforts.

## EC Regeneration Likely Involves More Than One Mechanism

Broadly, there are at least three major types of ECs in mammalian systems, related to arterial, venous, and lymphatic vessels (Coultas et al., 2005; Adams and Alitalo, 2007; Park et al., 2013; Qiu and Hirschi, 2019). These mature ECs are known to be arrested at the G_0_-phase of the cell cycle, and they have a limited turn-over rate, cycling once every 3–5 years *in vivo*. Thus, ECs are considered terminally differentiated cells. In principle, ECs could regenerate from adult EC stem cells; however, there is conflicting evidence regarding whether adult EC-stem cells actually exist *in vivo*. Many studies suggest the existence of adult hemangioblast and or angioblast, on the basis of CD34 and Flk1 expression (and other stem cell markers, e.g., Brachyury and Er71/Etv2), and the ability of these cells to form tube-like structures (Asahara et al., 1997; Loges et al., 2004; Hirschi, 2012). On the contrary, others have argued that these cells are likely to be present in low numbers in adults *in vivo* (Rafii, 2000; Park et al., 2013; Yoder, 2018; Qiu and Hirschi, 2019). Critiques have noted that bone marrow-derived monocytes and macrophages might have been misidentified as endothelial progenitor cells, thus confusing even the experts (Medina et al., 2017). However, genetic lineage tracing experiments in mice remain inconclusive regarding the presence of EC-stem cells. Recent article summarized the proangiogenic benefit observed in preclinical and clinical studies from over 700 patients in clinical trials of CD34 + cell therapy (Sietsema et al., 2019). Nevertheless, developmental studies suggest that venous ECs can be derived from arterial ECs, whereas lymphatic ECs can be derived from venous ECs (Wang et al., 1998; Yang et al., 2012). However, depending on the type of injury or damage experienced by the ECs, more than one mechanism is likely to activate EC regeneration. Our own studies have suggested that ECs become proliferative after experimental ischemia or myocardial infarction (Kohler et al., 2014; Baruah et al., 2017). Another mechanism is dedifferentiation followed by redifferentiation of ECs in the aftermath of ischemia, a process that can also be activated by administration of low-dose small molecule inhibitors of GSK-3b called BIO (6-bromoindirubin-3-oxime) and tideglusib/NP12 (Kohler et al., 2014; Baruah et al., 2017). Yet another mechanism might be the endothelial to mesenchymal transition (EndoMT) (Dejana and Lampugnani, 2018), a biological process that occurs during the formation of cardiac valves and contributes to the emergence of several other cell lineages (Monaghan et al., 2016), and is also a response to ischemia (Manavski et al., 2018).

Thus, it is reasonable to hypothesize that exosome-mediated regeneration of ECs is likely to include at least three distinct mechanisms, but not limited to:

• Exosomes that induce EC proliferation and survival, e.g., vasculogenesis and angiogenesis.
• Exosomes that induces EC dedifferentiation/redifferentiation (not a proven mechanism): for example, exosomes that upregulate Cyclin-D1 and down-regulate p53, p21, and p27 mRNAs should induce EC-dedifferentiation and rapid cell cycle progression.
• Exosomes that mediate EndoMT (not a proven mechanism); in principle, exosomes containing microRNAs (miRNAs) that downregulate VE-cadherin and up-regulate Twist, Slug and Snail, and matrix metalloproteases (MMPs) could mediate EndoMT.

Thus, genomic and non-genomic cargoes in exosomes that are capable of inducing signaling to one of the above events should provide EC regenerative benefit. In addition, regeneration of ECs might be possible via exosomes that mediate transdifferentiation of somatic cells or by directly reprogramming somatic cells into ECs.

In support of this idea, a few groups have addressed the possibility of using exosome mediated reprogramming of ECs for vascular regeneration (Cheng et al., 2017; Lee et al., 2017). For example, exosomes secreted by tumor cells carry a number of potent pro-angiogenic factors such as VEGF, TGFβ, bFGF, MMP2, and MMP9, mediated angiogenic activities of ECs (Skog et al., 2008; Giusti et al., 2016; Ludwig and Whiteside, 2018). This idea is currently being explored further in several laboratories in the settings of cardiovascular regeneration and rejuvenation. However, it remains to be seen if the exosome(s) mediated reprogrammed ECs have the ability to repair effectively and reestablish blood supply productively, in aftermath of ischemic episodes.

## Exosomes With Non-Genomic Cargoes That Mediate EC Regeneration

Myocardial infarction represents a major cause of death among all cardiovascular diseases. Injured cardiac tissues due to myocardial infarction or ischemic insult trigger a series of adaptive response, to initiate and drive repair the injured heart. Therefore, it was surmised that in the aftermath of myocardial infarction the injured myocardium might release extracellular vesicles and exosomes that could induce a regenerative program. Cardiac extracellular vesicles or exosomes are now known to be present in both normal and infarcted heart (Chistiakov et al., 2016). Therefore, these exosomes that are secreted in an infarcted heart mediate various cell to cell communication events, including exosome biogenesis which provide cardiovascular regenerative benefits, improved cardiac function, and normalize tissue homeostasis (Barile et al., 2012; Waldenström et al., 2012; Wang et al., 2016). In a study, human pediatric cardiac progenitor cell (CPCs) prepared from the right atrial appendages from children of different ages undergoing cardiac surgery for congenital heart defects were isolated and cultured under hypoxic or normoxic conditions. In their, study, the authors found that CPC exosomes derived from neonates improved cardiac function, mediated angiogenesis, and reduced fibrosis, independent of culture oxygen levels (Agarwal et al., 2017). However, there are many open questions that need to be addressed (Bollini et al., 2018). A detailed overview of exosomes and their regenerative potential in infarcted heart can be found elsewhere (Bollini et al., 2018; Shanmuganathan et al., 2018).

At the cellular level, EC proliferation and survival represent two key events in the process of EC regeneration (Park et al., 2013; Qiu and Hirschi, 2019). These cells must proliferate rapidly and survive to make up for the loss of cells or to replace damaged and non-functional cells. Inadequate proliferation or enhanced cellular death might initiate or augment pathological event. Therefore, well-coordinated cellular proliferation and survival events are quintessential to normalizing damaged tissues (McDonald et al., 2018).

A study on exosome cargo and EC interaction has been conducted by Nazarenko et al. (2010) in a tumor microenvironment. This study has described a role of Tetraspanin (Tspan8) containing exosomes, which efficiently induce angiogenesis in tumors and tumor-free tissues. The authors have found that Tspan8 contributes to selective recruitment of proteins and mRNAs into exosomes; these markers include CD106 and CD49d, which have been implicated in exosome-EC binding and EC internalization. Exosome uptake induces vascular endothelial growth factor (VEGF)–independent regulation of several angiogenesis-related genes, including *von Willebrand factor, Tspan8*, the chemokines *CXCL5*, and *MIF*, the chemokine receptor *CCR1* and, together with VEGF, *VEGF receptor 2* (Nazarenko et al., 2010). EC uptake of Tspan8-CD49d complex–containing exosomes is accompanied by enhanced angiogenic activities of EC, such as proliferation, migration, and sprouting. Several studies subsequently exploited the potential of exosome cargoes in a tumor-free environment. Accordingly, one elegant investigation by Sahoo et al. (2011) has shown that exosomes derived from human CD34^+^ stem cells mediate EC proliferation and survival, thereby stimulating the angiogenic activities of ECs. As expected, exosomes purified from human induced pluripotent stem cells have been found to induce angiogenesis and improve recovery in a mouse model of hind limb ischemia (Hu et al., 2015). Bian et al. (2013) have examined the effects of mesenchymal stem cell (MSC) derived extracellular vesicles which also included exosomes, and found that exosomes mediated efficient regeneration of ECs in a rat model of acute myocardial infarction. Although this study did not conclusively identify the types of molecules involved in this process, it highlighted the potential role of exosomes in mediating angiogenic processes in an injured tissue microenvironment (Shabbir et al., 2015; Teng et al., 2015). The Wnt/b-catenin signaling pathway is crucial in regulating both developmental and therapeutic angiogenesis (Dejana and Kühl, 2010). Interestingly, MSC exosomes express Wnt4, which induces translocation of (β-catenin into the nuclei of recipient ECs, thereby promoting angiogenic events in a rat skin burn model (Zhang et al., 2015). Similar studies have demonstrated that the Sonic hedgehog signaling pathway, the presence of platelet derived growth factor receptor in the extracellular vesicles or PKA signaling might contribute to the proangiogenic activity (Benameur et al., 2010; Ma et al., 2017; Xue et al., 2018). In a study, cardiomyocyte derived exosomes containing heat shock protein (Hsp20) showed increased EC proliferation by interacting with VEGF receptor-2 (Zhang et al., 2012). This finding highlighted the key role of exosomes in tissue restorative processes. Notch-Dll4 signaling has been extensively studied in relation to angiogenesis, whereby the expression of Dll4 ligand in tip cells regulates the sprouting of ECs (Gerhardt et al., 2003; Kangsamaksin et al., 2014; Pitulescu et al., 2017). In a 3D matrix microenvironment, exosomes containing Dll4 freely moved to target ECs and mediated efficient Notch activation upon interaction with the recipient ECs (Sharghi-Namini et al., 2014). In addition, Dll4-containing exosomes increased EC motility while decreased proliferation. Dll4 is known to be present during tissue reparative processes, and targeting Dll4 will be critical to mediating efficient angiogenic recovery in injured tissues. Angiogenesis is also regulated by the activities of MMPs, which mediate cell-matrix or cell-cell interaction during the migratory phase. In this context, MMP14 containing exosomes have been shown to cleave VEGFR1 and promote VEGF-A induced migration and proliferation of ECs (Han et al., 2019).

**FIGURE 1:**
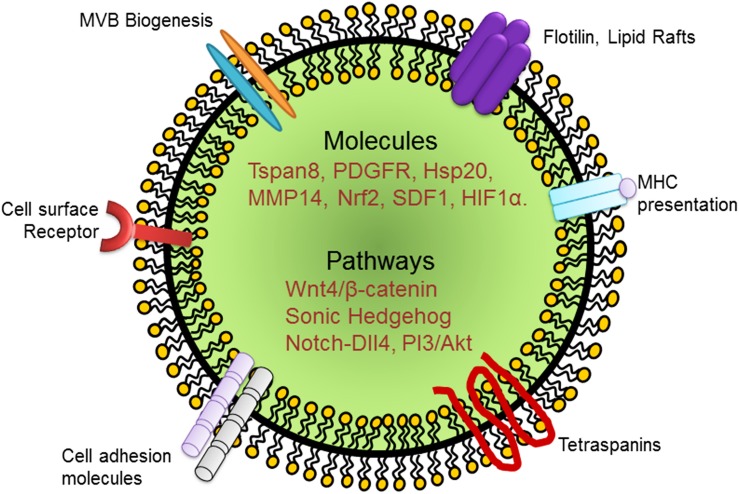
Schematic representation of an exosome and its cargo carrying growth factors, cytokines, and signaling molecules that have been implicated in EC regeneration. PDGFR, platelet derived growth factor receptor; Hsp, heat shock protein; MMP, matrix metalloprotease; SDF, stromal derived factor; HIF, hypoxia inducible factor.

Ding et al. (2019) have studied the effects of exosomes derived from bone marrow MSCs and found that they have superior angiogenic properties and enhance cell proliferation. The authors additionally found that deferoxamine conditioned exosomes activate the PI3/AKT pathway, thereby enhancing cell proliferation and decreasing wound lesions (Ding et al., 2019). Interestingly, exosomes derived from MSCs released high levels of the proangiogenic molecule stromal cell derived factor 1 (SDF1), which not only prevented apoptotic cell death of myocardial cells but also induced cardiac EC regeneration in a mouse model of myocardial infarction (Gong et al., 2019). Hypoxia inducible factor-1α (HIF-1α) is an important mediator of angiogenic activity during ischemic insult. Exosomes prepared from human umbilical cord MSCs have been found to enhance fracture repair and angiogenesis in a rat model of stabilized fracture through HIF1α (Zhang et al., 2019). Beyond studies of heart and skin injury models, the ability of exosome mediated regeneration has also been tested in a mouse model of traumatic brain injury (Gao et al., 2018). Here, the authors addressed the role of exosomes derived from endothelial colony forming cells in their ability to restore the blood brain barrier continuity (Gao et al., 2018). However, whether exosomes can also mediate EC regeneration via EndoMT in addition to the above mentioned mechanisms remain incompletely understood. Figure 1 and Table 2 summarize some of the key genomic and non-genomic cargoes implicated in the regeneration of ECs and angiogenesis.

**TABLE 2:** Non-genomic and genomic exosomal constituents and their known endothelial cell activities.

**Cellular activity**	**Cargo**	**References**
**EC proliferation and survival**	**Non-genomic**	
	• Tetraspanin (Tspan8)	Nazarenko et al., 2010
	• Wnt4	Zhang et al., 2015, 4
	• PDGFR	Ma et al., 2017
	• Sonic hedgehog pathway	Benameur et al., 2010
	• Protein kinase A signaling	Xue et al., 2018
	• pathway	Zhang et al., 2012
	• Heat shock protein (Hsp20)	Sharghi-Namini et al., 2014
	• Notch-Dll4	Han et al., 2019
	• MMP14	Ma et al., 2017
	• Nrf2	Gong et al., 2019
	• SDF1	Zhang et al., 2019, 1
	• HIF1α	

**EC proliferation and survival**	**Genomic**	
	• miRNA-146a	Ibrahim et al., 2014
	• miRNA-294	Khan et al., 2015
	• miR-21-3p	Hu et al., 2018
	• miR-939	Li et al., 2018
	• miR-423-5p	Xu et al., 2019
	• miR-210	Ma et al., 2018
	• miR-199-5p	Ye et al., 2019)

*EC, endothelial cells.*

## Exosomes With Genomic Cargoes That Mediate EC Regeneration

**FIGURE 2:**
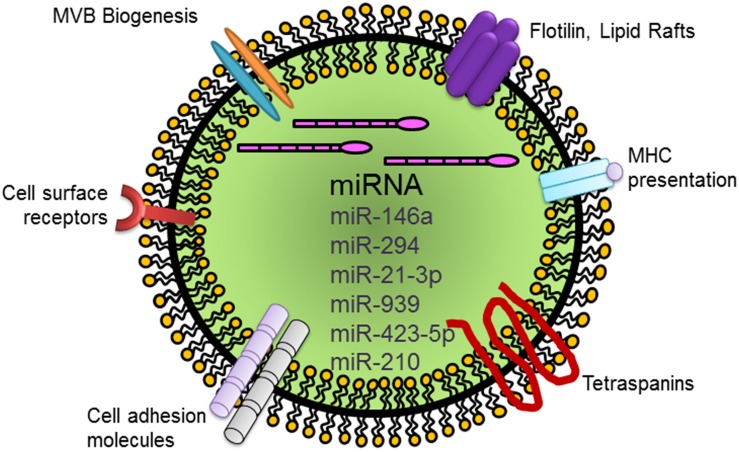
Schematic representation of a generic exosome and its cargo carrying miRNAs that mediate EC regeneration. Abbreviation: miRNA, microRNA.

In addition to transporting growth factors and receptors, exosomes possess the unique ability to transfer miRNAs to recipient cells. miRNAs regulate downstream signaling events through base pairing of their seed sequence with complementary mRNA (Lu and Clark, 2012). MSCs, as well as ECs, contain different regulatory miRNAs, which alter cellular function in target cells (Hromada et al., 2017; Ferguson et al., 2018). In this context, miRNA-146a enriched exosomes secreted from cardiosphere-derived cells have been shown to enhance angiogenesis while simultaneously stimulating proliferation and inhibiting apoptosis of cardiomyocytes (Ibrahim et al., 2014). Exosomes derived from embryonic stem cells have also been exploited in this regard. Accordingly, mouse ESC-derived exosomes have been shown to provide beneficial effects in regeneration after myocardial injury via miR-294 (Khan et al., 2015). Different miRNAs have been implicated in this reparative process. In a more recent study, miR-21-3p enriched exosomes secreted by human umbilical cord blood cells have been shown to accelerate cutaneous wound healing and promote angiogenic events (Hu et al., 2018). Yet another interesting study conducted in a patient population with myocardial ischemia has reported that coronary serum exosomes regulated angiogenesis through miR-939 in this sample group (Li et al., 2018). Human adipose derived stem cell exosomes also exert similar proangiogenic effects via miR-423-5p and Sufu (Xu et al., 2019). In addition, exosomes loaded with miR-210 exert beneficial effects favoring EC function and reoxygenation (Ma et al., 2018). Thus, exosomes can transport different combinations of miRNAs depending on the tissue environment and cell type (Kim et al., 2012; Ferguson et al., 2018). We have listed a select group of miRNA cargoes transported by exosomes known to regulate EC regeneration and angiogenesis in Figure 2 and Tables 2, 3. Nevertheless, continued analyses of the miRNA compositions of various exosomes should be useful in designing custom exosomes for the induction of potent EC regeneration in relation to angiogenesis and revascularization of ischemic cardiovascular tissues. A complete understanding of the role of exosomes in regenerative process in the aftermath of myocardial infarction could bridge an important gap in knowledge of the repair mechanism after myocardial injury.

**TABLE 3:** Exosomes in endothelial cell proliferation and angiogenesis.

**Key findings**	**References**
Improved angiogenesis in rat hind limb ischemia model	Johnson et al., 2019
Promoted EC repair in a rat model of balloon –induced carotid artery injury	Hu et al., 2019
Enhanced repair effect in a rat model of myocardial infarction	Ni et al., 2019
Promoted postnatal angiogenesis in mice bearing ischemic limbs	Ye et al., 2019
Human induced-pluripotent stem cell-derived cardiomyocytes promoted angiogenesis	Dougherty et al., 2018
Exosomes derived from ischemia subjected cardiomyocytes promoted cardiac angiogenesis	Ribeiro-Rodrigues et al., 2017
Human pericardial fluid derived exosome promoted therapeutic angiogenesis	Beltrami et al., 2017
Enhanced the density of new functional capillary and blood flow recovery in rat myocardial infarction model	Teng et al., 2015

## Future Perspectives

The capacity of the exosomes to induce EC regeneration should benefit organ repair and survival after injury. EC regeneration and the ways in which therapeutic exosomes contribute to this process have the potential in treating ischemic cardiovascular diseases. Thus, substantial progress has been made in the field of exosome research, providing insights into exosome composition and function. Ongoing methodological and technical innovations are beginning to help further synthesize new knowledge, functional understanding and potential applications. However, detail studies are needed to address the possible heterogeneity of exosomes and how this new knowledge could benefit the understanding of EC regeneration and EC pathology. For example, are there specific stimuli that induce the release of “exosomes” that mediate angiogenic activities of ECs, but do not alter the behavior of any other cell type? Are there exosomes that induce rapid proliferation of ECs, but not non-ECs? Are there specific exosomes that inhibit fibrosis, but induce productive wound healing in the aftermath of acute myocardial infarction? These are some of the few questions that come to mind as we ponder the future of exosomes in applications in EC regeneration and re-establishing blood flow to the ischemic cardiovascular organs. Studies have attempted to determine the regenerative ability of exosomes primarily in inbred (e.g., C57BL/6) mouse strains, in experiments such as hind limb ischemia and myocardial infarction. Usually, C57BL/6 mice show robust EC regenerative activities. The question remains whether exosomes provide potent EC regenerative responses in a strain-specific manner. Experiments are also needed in clinically relevant models, for example, mice with defective revascularization potential, such as diabetes. Unraveling the molecular and functional attributes of exosomes and how they may be harnessed should contribute meaningfully to the pursuit of controlling the biology of ECs for regenerative therapy. The answers to these questions and concerns should arrive soon, as new technological innovations such as organoids, data science, computational modeling and artificial intelligence are being incorporated into cardiovascular research (Garikipati et al., 2018; Trac et al., 2019).

## Conclusion

In principle, more than one mechanism is likely to be involved in regulating EC repair and regeneration, and reestablishing flow of blood to the ischemic organs. However, there are technical challenges that must be addressed before exosomes could be used as therapeutic biologics from bench to bedside. In this review, we have attempted to summarize how the cargo composition of exosomes derived from several human and non-human sources might benefit EC repair and regeneration. It would be a “giant leap” to be able to reprogram autologous somatic cells directly to ECs by using exosomes, thereby eliminating the use of viral vectors. However, continued research will be required before this interesting idea can be translated into therapy through EC regeneration and restoration of cardiovascular function.

## Author Contributions

Both authors wrote, corrected, and reviewed the final manuscript. KW submitted the manuscript online.

## Conflict of Interest

The authors declare that the research was conducted in the absence of any commercial or financial relationships that could be construed as a potential conflict of interest.
